# Bridging the gaps in test interpretation of SARS-CoV-2 through Bayesian network modelling

**DOI:** 10.1017/S0950268821001357

**Published:** 2021-06-23

**Authors:** Yue Wu, David Foley, Jessica Ramsay, Owen Woodberry, Steven Mascaro, Ann E. Nicholson, Tom Snelling

**Affiliations:** 1 School of Public Health, University of Sydney, Camperdown, New South Wales, Australia; 2 Department of Infectious Diseases, Perth Children's Hospital, Perth, Western Australia, Australia; 3 Wesfarmers Centre for Vaccines and Infectious Diseases, Telethon Kids Institute, Perth, Western Australia, Australia; 4 Department of Data Science & Artificial Intelligence, Monash University, Clayton, Victoria, Australia; 5 School of Public Health, Curtin University, Bentley, Western Australia, Australia; 6 Menzies School of Health Research, Charles Darwin University, Darwin, Northern Territory Australia

**Keywords:** Bayesian belief model, causal diagram, diagnostic decision support, false negative, RT-PCR test, SARS-CoV-2

## Abstract

In the absence of an established gold standard, an understanding of the testing cycle from individual exposure to test outcome report is required to guide the correct interpretation of severe acute respiratory syndrome-coronavirus-2 reverse transcriptase real-time polymerase chain reaction (RT-PCR) results and optimise the testing processes. Bayesian network models have been used within healthcare to bring clarity to complex problems. We use this modelling approach to construct a comprehensive framework for understanding the real-world predictive value of individual RT-PCR results.

We elicited knowledge from domain experts to describe the test process through a facilitated group workshop. A preliminary model was derived based on the elicited knowledge, then subsequently refined, parameterised and validated with a second workshop and one-on-one discussions.

Causal relationships elicited describe the interactions of pre-testing, specimen collection and laboratory procedures and RT-PCR platform factors, and their impact on the presence and quantity of virus and thus the test result and its interpretation. By setting the input variables as ‘evidence’ for a given subject and preliminary parameterisation, four scenarios were simulated to demonstrate potential uses of the model.

The core value of this model is a deep understanding of the total testing cycle, bridging the gap between a person's true infection status and their test outcome. This model can be adapted to different settings, testing modalities and pathogens, adding much needed nuance to the interpretations of results.

## Introduction

Effective containment of coronavirus disease-2019 (COVID-19) caused by the severe acute respiratory syndrome coronavirus 2 (SARS-CoV-2) rests upon the rapid and accurate identification of cases. Although nucleic acid amplification tests, including real-time reverse transcriptase polymerase chain reaction (RT-PCR), are widely used, the absence of an established gold standard diagnostic method has hindered the assessment of test performance [1]. The potential for false-negative results is well-recognised; such results can significantly undermine the public health response, facilitating ongoing chains of transmission. Similarly, at the patient level, it may delay case recognition, place other patients and healthcare workers at risk and, importantly, impede the commencement of emerging treatments. A wide variation in rates of false negatives has been reported, ranging from 1.8 to 58% [2]; this variability may be attributable to heterogeneity in disease prevalence, patient age, timing of testing, type of specimen, other components of the pre-analytical phase and the RT-PCR assay employed across studies [3].

Although better standardisation of data collection and reporting may add further clarity, a comprehensive understanding of the mechanisms involved in testing is required to help develop strategies to improve testing systems and importantly, guide the correct interpretation of test results within the associated context. This includes a true understanding of the positive and negative predictive values of a test at a national, regional and patient level and the potential to permit the early identification of false-negative results. The limitations in available data undermine these efforts. An alternative method is required to bridge the current gaps in knowledge.

Bayesian network (BN) modelling offers an approach to understanding complex problem domains by organising information, whether directly observable or not (i.e. latent), under a causal inference framework [4, 5]. A BN is a probabilistic graph model that integrates available data with subject-matter knowledge from domain experts to describe how a system operates [6]. BNs have been used within healthcare to improve clinical decision-making [7], bringing clarity to complex problems, especially where there is little quality data available [7]. As a pertinent example, Fenton and colleagues have highlighted the need for causal models and showed how different sampling methods, testing and reporting procedures may contribute to the variation in observed COVID-19 death rates among countries under a BN framework [8]. We elicited knowledge from a range of domain experts to construct a causal BN which describes the testing process for SARS-CoV-2 by RT-PCR, from individual exposure through to the interpretation of the laboratory test result. In explicitly modelling the latent trajectory of a pathogen through its diagnostic pathway, we have generated a common framework which accounts for a range of factors that plausibly influence test results, and which may be generalisable to other pathogens and assay formats. This model requires validation with local, real-world datasets prior to application. We intend for future applications to integrate with other models that detail local epidemiological factors such as those developed by Fenton *et al*. [8] to account for the complex and dynamic interactions between individual-level factors (e.g. gender [9] and comorbidities) and population-level behaviours (e.g. public health policies and population behaviours) that influence the transmission and prevalence of SARS-CoV-2.

## Methods

A BN comprises two parts: (1) a graph that uses nodes to represent the factors (or variables) which are relevant to describing and understanding the system, and arrows to represent the direct statistical (and often causal) dependencies between them; and (2) a set of conditional probability distributions that specifies the strength of each of those dependencies, which then forms a joint probability distribution over all variables.

Clinical experts in microbiology, infectious diseases, epidemiology, public health and general medicine contributed their relevant subject matter knowledge. Key variables were identified through literature review and an online discussion with the experts. Knowledge elicitation was guided by trained facilitators, defined as knowledge engineers, and supported utilising graphical representations of interactions between variables within the proposed structure [6, 10]. A preliminary model structure was created via a subsequent group knowledge elicitation workshop; this was then reviewed and refined in one-on-one discussions with the experts. A preliminary parameterisation of the model was performed to produce qualitative behaviour that matched the modellers' and the experts' high level understanding of the problem domain. The refined model structure was reviewed and validated by experts in a second workshop and one-on-one discussions.

We provide a narrative description of the model structure and illustrate its potential application in four scenarios. All nodes are labelled and referred to by numbers ***1–31***. The term ‘virus’ and all described events relate to SARS-CoV-2, unless stated otherwise. The model was built in GeNIe (https://www.bayesfusion.com/downloads/). Appendix A provides a comprehensive variable dictionary for this model, with references to justify each node and arc where possible. Detailed conditional probability tables can be accessed via the OSF platform, which will also include any future updates.^1^

## Results

**Fig. 1.**
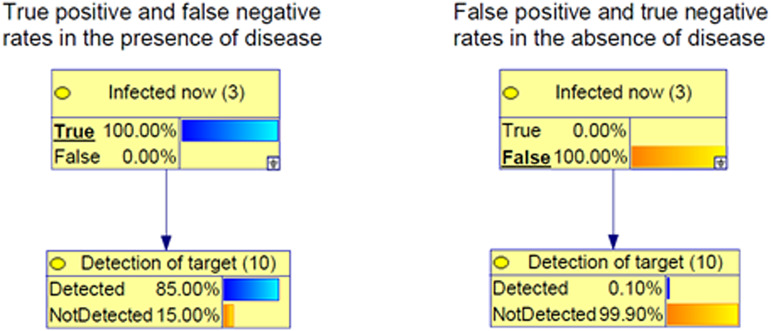
Definitions of true and false positives and negatives for laboratory results. (Left) The true positive rate is the probability of Detected amongst those infected and the false-negative rate is the probability of NotDetected also amongst those infected. (Right) The false-positive rate is the probability of Detected amongst those who are not infected and the true-negative rate is the probability of NotDetected also amongst those who are not infected.

Figure 1 shows the simplest possible BN for representing true and false-positive and -negative rates produced by laboratory results.^2^ The figure illustrates this simple BN in two scenarios: when a person is infected now (left) and when a person is not infected now (right). According to this BN, there is an 85% chance of detecting the virus if a person is infected at the time of testing, giving a corresponding false-negative probability of 15%; and a 0.1% chance of falsely detecting the virus if a person is not infected, giving a corresponding 99.9% probability of a true negative. Obtaining accurate estimates of these rates is challenging because we cannot directly observe (nor perfectly control) the true infection status at the time of testing – the very reason a test is needed – and hence must make do with general estimates based on controlled samples. We can, however, make improved case-specific estimates by incorporating factors involved in the process of sampling and testing into our model. We can also improve our assessment of whether a person is truly infected by incorporating background factors (such as age) that may influence both the prior probability of an infection as well as other factors related to the testing process (such as the chance of finding virus at a particular sample site). The model we present next describes how we have expanded this simple model to include other relevant variables that interact to drive changes to sensitivity, specificity and, ultimately, the probability of infection.

### Model description

**Fig. 2.**
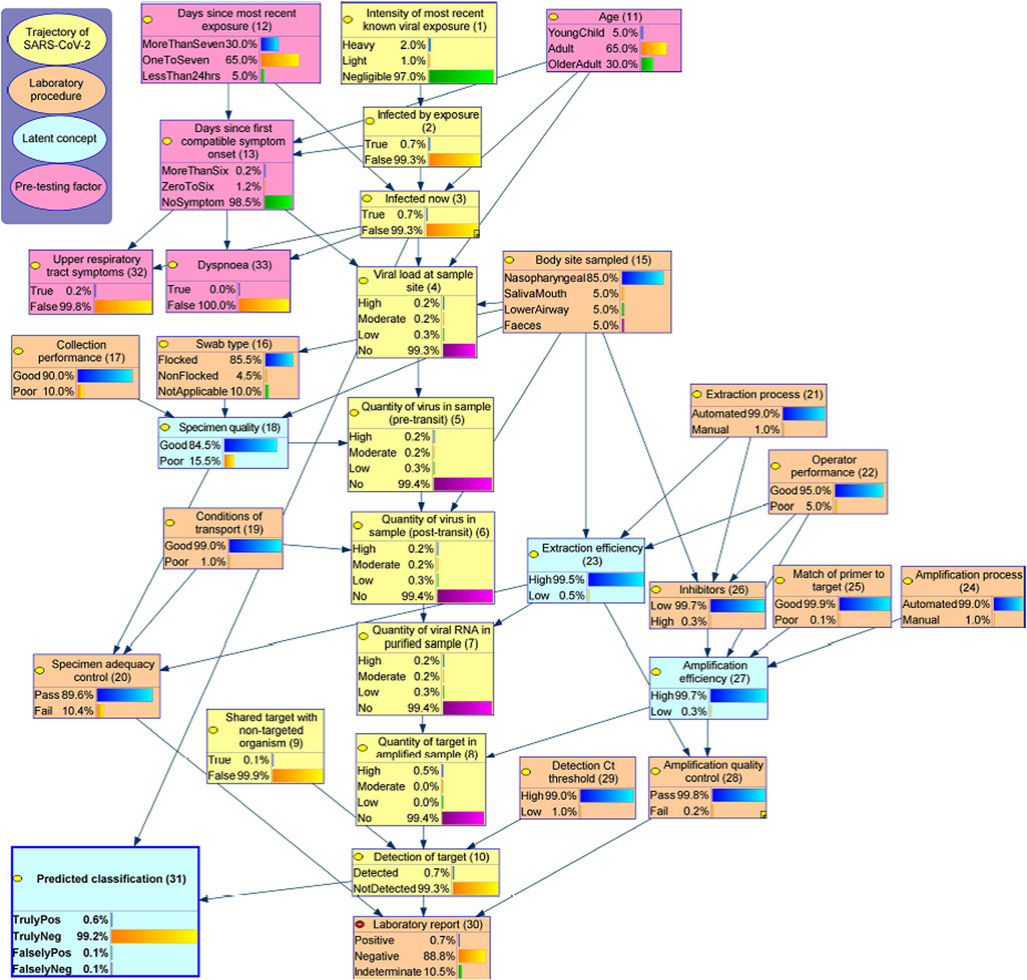
The causal BN of RT-PCR testing of SARS-CoV-2. This diagram presents the model structure, variable values and marginal distributions (i.e. when nothing is known, other than that a test has been conducted). Appendix A provides a comprehensive variable dictionary for this model. Detailed conditional probability tables can be accessed via Appendix B_1.

The expanded BN (Fig. 2) models the trajectory of SARS-CoV-2 as it's sampled, transported, extracted and amplified, along with the conditions and operations that can affect the sample throughout this process.^3^ The SARS-CoV-2 trajectory itself is modelled via a sequence of latent nodes (coloured yellow, ***1–10***), running down the centre of the graph, with the previously introduced **infected now** (***3***) and **detection of target** (***10***) sitting at almost opposite ends of this sequence. The probability of being **infected by a known viral exposure** (***2***) is driven by the **intensity of that exposure** (***1***). If infected by the known exposure (***2***), **age** (***11***) and the number of **days since the exposure** (***12***) influence the probability of being **infected at the time of testing** (***3***) and also drive the **days since first compatible symptom onset** (***13***). **Upper respiratory symptoms (*32*)** and **dyspnoea (*33*)** are used to illustrate manifestation of disease severity. The probability of infection from an unknown exposure is possible and currently parameterised to be low, although this risk is influenced by the background prevalence of the virus in a given population at a given time, and therefore needs to be calibrated according to the setting. The background probability of compatible symptoms unrelated to a known exposure (potentially non-SARS-CoV-2) is also set to be low, and as a result, the presence of symptoms predicts a high probability of being infected by a recent exposure. However, this background probability is also driven by the circulation of other symptom-compatible pathogens.

Among those with **SARS-CoV-2 infection at the time of testing** (***3***), the **viral load at a given sample site** (***4***) is influenced by the number of **days since first symptom onset** (***13***),^4^ **body site sampled** (***15***) and **age** (***11***). In particular, the model assumes the viral load in the upper airway is initially highest, followed by increasing amounts in lower respiratory tract and faeces over time.

When collecting a sample, the quantity of virus obtained from the **viral load at sample site** (***4***), equating to the **quantity captured in sample (pre-transit)** (***5***) depends on the **specimen quality** (***18***), a latent variable which captures the technical and operator-dependent factors which affect the adequacy of collection. **Specimen quality** (***18***) is therefore improved by a good **collection performance** (***17***) (e.g., indicated by collector's experience), the use of a flocked **type of swab** (***16***) (if applicable) and a site that requires a simpler collection technique, such as **specimen sampled from** saliva and mouth sites (***15***). The **quantity of virus in sample post-transit** (***6***) may be affected by **the quantity of virus in the sample pre-transit** (***5***), the **conditions of transport** (***19***) and **body site sampled** (***15***); for example, faecal specimens may contain substances which accelerate the degradation of viral nucleic acid.

In the laboratory, the **extraction** and **amplification processes** (***21***, ***24***) are assumed to be predominantly automated, meaning a testing process that is less affected by **operator performance** (***22***), compared with manual methods. In addition, a high level of **inhibitors** (***26***) and a poor **match of primer to target** (in the virus) (***25***) can reduce **amplification efficiency** (***27***). Low **extraction** and **amplification efficiency** (***23***, ***27***) (both latent) may increase the probability of a false negative result if the **quantity of virus** is low in the **post-transit** (***6***) and **purified samples** (***7***), respectively. The probability of a false-negative result may also increase if the **detection Ct threshold** (***29***) is lowered (e.g. to 35). A false-positive result may occur if the specimen contains a **shared target from a non-SARS-CoV-2 organism** (***9***). Similarly, if the **detection Ct threshold** (***29***) is set higher (e.g. 40 and above), the risk of non-specific amplification increases and, consequently, the risk of a false-positive result. These events are assumed to be rare by the current model.

Finally, the **lab report** (***30***) will be detected if the **viral target is detected** (***10***), often described as a positive result. If the target is not detected, the test result will be reported as not detected if the specimen has passed both the **specimen** and **amplification quality controls** (***20***, ***28***) (a negative result) and as indeterminate otherwise (where a repeat test may be requested). In cases where the **SARS-CoV-2 target** (***10***) is *not detected*, there is a high likelihood that this represents a true or false-negative result if the probability of being **infected now** (***3***) is low or high, respectively, and likewise for true or false positive. This relationship is now described using the node **predicted classification** (***31***). It is worth noting that this node reports the *marginal* probability of any classification being *truly positive*, *truly negative*, *falsely positive* or *falsely negative* and corresponds to the (predicted and normalised) confusion matrix. Thus, the probability of a *truly positive* case is the joint probability that someone is both infected (i.e. positive) and classified as positive. It should be kept in mind that this differs from the definition of the true positive rate, and other rates, which are *conditional* on being *truly infected* (as described in Fig. 1).

### Example scenarios

Four illustrative scenarios were developed in conjunction with the experts. The model outputs were obtained by setting the input variables as ‘evidence’ for a given constructed scenario. Appendix B can be accessed for detailed models with input variables selected for each scenario. Scenarios 1–3 assume a setting with low viral prevalence in the population (0.1%) and Scenario 4 compares the low prevalence setting with a high prevalence setting (5%).^5^

#### *Scenario 1:* The predicted probability of infection and testing results influenced by exposure intensity and presence of symptoms

Consider an **older adult** (***11***) who had a **light exposure** (***1***) to the virus **1−7 days ago** (***12***) (e.g. brief contact in a cafe) but with **no symptoms** (***13***) currently. The probability of this person **currently being infected** (***3***) is estimated to be 1.1%, and the probability of returning a **positive nasopharyngeal swab** (***15***) result is 1.0% (***30***) with predicted 0.2% chance of a **falsely negative result** (***31***). However, if the **intensity of exposure was heavy** (***1***) (e.g. household contact), the risk of **being infected** (***3***) would be 7.9% and the probability of returning a **positive test result** (***30***) would be 6.4% with **falsely negative classifications** occurring 1.6% of the time (***31***). Rather than having no symptoms, if the person experienced onset of **symptoms 0−6 days** (***13***) **after that exposure** (***1***), the probability of **being infected** (***3***) is estimated to be 96.5%, and the probability of returning a **positive result** (***30***) is 92.3% (with **falsely negative predictions** (***31***) occurring 4.2% of the time).

#### *Scenario 2:* Influence of specimen quality on the probability of a positive test result in those who are infected

Consider the same **older adult** (***11***) who was **heavily exposed** (***1***) **to the virus 1−7 days ago** (***12***) and had onset of **symptoms 0−6 days afterwards** (***13***)**.** Consider now that this person **is infected** (***3***). A **nasopharyngeal swab** (***15***) is taken for testing. If a **poor collection** (***17***) is performed with a **non-flocked swab** (***16***) and the **conditions of transport** (***19***) are poor, the probability that the lab reports a **positive result** (***30***) is 89.2%, the probability of an indeterminate result is 6.5%, and the probability of a falsely negative result (***31***) is 10.8%. However, for a **good collection performance** (***17***) using a **flocked swab** (***16***) and with **good specimen transport conditions** (***19***), the probability of a **positive result** (***30***) is 95.9%, the probability of an indeterminate result is 0.6% and the probability of a falsely negative result (***31***) is 4.1%.

#### *Scenario 3:* Understanding the patient characteristics of those with false-negative test results

Individuals who are truly **infected at the time of testing** (***3***) but who **tested negative** (***30***) (i.e. false negatives) are **younger** (***11***) and have a higher probability (69.8%) of having **no symptom at the time of testing** (***13***) than those who are infected and **test positive** (***30***) (27.6%).

#### *Scenario 4:* Significant impact of baseline prevalence of disease on false-negative rates

In the model, the baseline prevalence of disease is simulated using parameters that describe the probability of being **infected now** (***3***) and the probabilities of experiencing **compatible symptoms** (***13***, ***32*** and ***33***), conditional on not being infected by known exposures. Comparing low and high prevalence (0.1% *vs.* 5% underlying incidence of the virus), for an individual who has **negligible known recent viral exposure** (***1***) and experiences **upper respiratory symptoms** (***32***) but **tested negative** (***30***), the probability of a *falsely negative* result is 0.3% and 14.6%, respectively. In the high prevalence setting, if the same person experienced dyspnoea (rather than upper respiratory symptom/s), the simulated *falsely negative* probability increases to 76.5%.

## Discussion

Accurate diagnosis of COVID-19 is critical to guide patient management, including infection control and public health responses [11]. Although there is increasing data on the performance of commercial assays [12], these assessments typically use non-clinical samples and are performed in closely controlled environments. A range of variables, from the age of the subject, the nature of exposure, the presence and duration of symptoms, operator skill and assay technical complexity can all influence the positive and negative predictive value of a test and are therefore important considerations when interpreting any test result. The core value of this model is its explicit representation of these variables and their probabilistic interdependencies, allowing a deeper understanding of test results by explicitly illustrating how truly positive or negative and falsely positive or negative cases can arise, based on the discrepancy between a person's true infection status and what leads to a particular test outcome. At a population level, the model demonstrates how different levels of local prevalence can significantly affect the interpretation of test results (illustrated in Scenario 4). It can also be used to identify how and where improvements in processes and procedures may improve the value of the test. Given a fully parameterised and calibrated model, tracking changes in the distribution of these variables (depicted in Scenario 3) over time and across settings can help understand how public health responses can be optimised for the timely detection of cases [11] so that effective containment strategies can be implemented.

When the model is applied to a single patient, it can also inform individual-level management. The probability that a person is infected can be more correctly inferred by integrating the test result with knowledge of the background risk (or ‘pre-test probability’), the intrinsic assay characteristics and the adequacy of the sampling and laboratory procedures. This is illustrated in Scenario 1 where there is still a 4.2% chance that a falsely negative result will be obtained in an older patient with infection and symptoms, which could have significant ramifications in terms of that patient's outcome and the risk of spread. Importantly, if a negative result was obtained, the model would allow this result to be reviewed in context, guiding the clinician's interpretation of the result through a better understanding of the negative predictive value for that individual patient.

Causal BNs allow the exploration and characterisation of a complex problem based on elicited knowledge from domain experts, even when limited data are available; a valuable characteristic during an outbreak of a novel pathogen. The model allows inclusion of known components of the testing cycle, including specimen collection and transport [13, 14], elements that are often not known when interpreting the result. Specimen adequacy can influence the amount of virus present at the site that is ‘collected’ for testing. Poor collection performance may reduce the advantage of the more technically difficult to collect specimen [15]. Scenario 2 underlines the importance of a good specimen collection, coupled with other factors, decreasing the probability of a falsely negative result from 10.9% to 4.1%. Similarly, although mouth and saliva swabs are technically easier to collect, better tolerated and may facilitate self-collection, they have a potentially lower predictive value due to the lower quantity of virus at that site [15].

The expanding drive for testing and pressure of rapid turnaround times places enormous strain on laboratory staff and testing systems. Although capacity has increased, the impact of the human element cannot be underplayed [16] and needs to be accounted for when considering the predictive value of a result; inexperienced staff, extended work hours and increased pressure can impact the numerous intricate steps of laboratory testing and thereby affect test performance. Automated processes can mitigate some potential errors, yet are not available in all settings across the globe. Laboratory quality assurance measures including extraction controls may help to identify systematic errors and reduce false negatives secondary to poor extraction or the presence of inhibitors.

COVID-19 RT-PCR assays have been designed to match the novel emerged virus. A future concern, included in the model, is the potential drift and divergence of COVID-19 strains into distinct lineages. These changes may alter the amplification site, reducing the RT-PCR ability to detect the presence of different lineages. Similar evolutionary changes have been observed in influenza, requiring re-tooling of the nucleic acid amplification [17].

The model can be calibrated to account for the changing population incidence of COVID-19 and adjusted for low or high viral incidence rates. Rates of co-circulating pathogens can also be incorporated into the model. For example, respiratory syncytial virus and influenza were low in Australia [18] during the winter of 2020; in the model this would increase the probability that respiratory symptoms after an exposure would be suggestive of COVID-19 infection. As the northern hemisphere enters their winter, the model can be calibrated to reflect their rates over their typical peak season. The model also has the flexibility to be modified based on application to account for the different performance of RT-PCR platforms used in different laboratories, as well as for other pathogens.

## Limitations

To guide individual and public health decision-making, the model will need to be validated using data. Expert opinion may incorrectly guide the model, as current knowledge and experiences may not be generalisable to this outbreak. Further setting-specific parameterisation and validation of the model is required before introducing into a real-world setting. These should involve important parameters such as location and population-specific prevalence of SARS-CoV-2 and other respiratory viruses with compatible symptoms, and the changing viral load at each sample site at the different time points post infection.

Simulated data that support the findings of this study are openly available on OSF at osf.io/t834y

## Footnotes

1 Link to OSF https://osf.io/t834y/

2 Node numbers have been kept consistent with the full network in Figure 2.

3 For specific variable definitions, see Appendix A, the variable dictionary.

4 The arrow from node (13) to node (4) is the only non-causal link in this model, it summarises events that occur during the time from first symptom onset to testing that may affect viral load at sample site (accumulation or decrease).

5 Please access models in GeNIe via OSF: https://osf.io/t834y/

## Supplementary material

For supplementary material accompanying this paper visit http://dx.doi.org/10.1017/S0950268821001357. click here to view supplementary material
