# Supplementary material for: Bridging the gaps in test interpretation of SARS-CoV-2 through Bayesian network modelling
Source: Epidemiol Infect. 2021 Jun 23;149:e166. doi: 10.1017/S0950268821001357 (PMC8314199; doi:10.1017/S0950268821001357)
Supplement: Supplementary file 1 [file hygsup.zip › S0950268821001357sup001.docx]

**Appendix A. Variable dictionary**

| **Variable name** | **Description** | **Potential values** | **Status** | **Parent nodes** | **References** |
| --- | --- | --- | --- | --- | --- |
| **Intensity of most recent known viral exposure (1)** | Intensity of most recent exposure to SARS-CoV-2. This variable can be influenced by many epidemiological factors not covered in this current model. | *Heavy,*  *Light,*  *Negligible* | Latent | None | (1, 2) |
| **Infected by exposure (2)** | Whether the person was infected due to the most recent exposure. | *True,*  *False* | Latent | Intensity of most recent known viral exposure |  |
| **Age (11)** | Age of person tested. | *YoungChild,*  *Adult,*  *OlderAdult* | Observable | None | (3, 4) |
| **Days since most recent exposure (12)** | On the day of testing, the number of days since most recent known exposure to SARS-CoV-2. | *MoreThanSeven,*  *OneToSeven,*  *LessThan24hrs* | Observable | None | (5-7) |
| **Days since first compatible symptom onset (13)** | On the day of testing, the number of days since the onset of first (compatible) symptom. It is unknown whether the intensity of exposure influences the timing of symptom onset, so no corresponding arc has been included. | *MoreThanSix, ZeroToSix, NoSymptom* | Observable | Days since most recent exposure, age, infected by exposure | (5, 7-9) |
| **Upper respiratory tract symptoms (32)** | On the day of testing, whether the testing person is experiencing any upper respiratory symptoms. | *True,*  *False* | Observable | Days since first symptom onset, infected now | (8, 10) |
| **Dyspnoea (33)** | On the day of testing, whether the individual is experiencing dyspnoea. | *True,*  *False* | Observable | Days since first symptom onset, infected now | (8, 10) |
| **Infected now (3)** | SARS-CoV-2 infection (at any body site) at the time of testing. If not infected by the most recent known exposure, the risk of being infected now is currently set to be 0.1%. This probability should be driven by the spatial and temporal prevalence of the virus in a given setting. | *True,*  *False* | Latent | Days since most recent exposure, infected by exposure, age |  |
| **Body site sampled (15)** | Body site swabbed or bodily fluid collected for SARS-CoV-2 testing. A non-uniform prior is used for this node, with the majority (85%) sample site set to nasopharynx. | *Nasopharyngeal, SalivaMouth,*  *LowerAirway,*  *Faeces* | Observable | None | (9, 11, 12) |
| **Viral load at sample site (4)** | Amount of SARS-CoV-2 at sample site. The CPT of this node may vary by site which interacts with the progression of disease. | *High,*  *Moderate,*  *Low,*  *No* | Latent | Infected now, days since first compatible symptom onset, age, body site sampled | (13-16) |
| **Swab type (16)** | Type of swab used to obtain SARS-CoV-2 sample. This variable is only applicable to the sites sampled by swab. | *Flocked, NonFlocked, NotApplicable* | Observable | Body site sampled | (17), (18) |
| **Collection performance (17)** | The resulting performance of the sample collection process. This may be influenced by various factors (not explicated), including the ability of the person collecting the sample for SARS-CoV-2 testing. | *Good,*  *Poor* | Observable | None | (19, 20) |
| **Specimen quality (18)** | This is a latent concept that summarises the quality of the specimen, collected for SARS-CoV-2 testing, in terms of its ability to confer an accurate result. | *Good,*  *Poor* | Latent | Collection performance, swab type, body site sampled | (6, 9, 11, 21-25) |
| **Quantity of virus in sample (pre-transit) (5)** | The number of viral particles obtained for testing within the sample. | *High,*  *Moderate,*  *Low,*  *No* | Latent | Viral load at sample site, specimen quality |  |
| **Conditions of transport (19)** | The conditions the specimen is subjected to, from collection to laboratory processing. This includes temperature, transport time, transport with/without viral transport media, which are observable but not explicitly described in the current model. | *Good,*  *Poor* | Observable | None | (9, 26) |
| **Quantity of viable virus in sample (post-transit) (6)** | The remaining amount of detectable virus in the sample after exposure to transport conditions. | *High,*  *Moderate,*  *Low,*  *No* | Latent | Conditions of transport, body site sampled, quantity of virus in sample pre-transit |  |
| **Extraction process (21)** | The process by which the nucleic acid is extracted from the collected sample. Extraction process can differ with manual and automated processes. Prior distribution sets 99% to be automated, however this might not be the case in resource-poor settings. | *Automated, Manual* | Observable | None | (9, 27) |
| **Operator performance (22)** | The accuracy of the scientist/technician performing the PCR testing procedures in the lab. In addition to experience, this may also be influenced by other factors, such as workload, which are not covered in the current model. | *Good,*  *Poor* | Observable | None | (28, 29) |
| **Extraction efficiency (23)** | The collective quality of the nucleic acid extraction process to produce a sample able to detected SARS-CoV-2 if present. | *High,*  *Low* | Latent | Body site sampled, extraction process, operator performance | (30, 31) |
| **Quantity of viral RNA in purified sample (7)** | The amount of SARS-CoV-2 viral RNA extracted from the sample. | *High,*  *Moderate,*  *Low,*  *No* | Latent | Quantity of virus in sample post-transit, extraction quality, operator performance | (9) |
| **Inhibitors (26)** | Any factor which prevents the amplification of SARS-CoV-2 RNA during the PCR testing of the sample. | *Low,*  *High* | Latent | Conditions of transport, body site sampled, extraction process, operator performance | (9, 32, 33) |
| **Amplification process (24)** | The process of replicating the SARS-CoV-2 RNA sequence(s) millions of times. Prior distribution sets 99% to be automated, however this might not be the case in resource-poor settings. | *Automated, Manual* | Observable | None | (30) |
| **Match of primer to target (25)** | The closeness in similarity of the nucleic acid sequence used in the PCR assay to the nucleic acid sequences in the extracted RNA from patient sample. Prior distribution sets 99.9% to be good match, especially as there is often more than one target selected for the primer. | *Good,*  *Poor* | Observable | None | (34-37) |
| **Amplification efficiency (27)** | The collective quality of the test sample after the amplification process. | *High,*  *Low* | Latent | Inhibitors, operator performance, match of primer to target, amplification process |  |
| **Quantity of target in amplified sample (8)** | The amount of RNA in the sample present after amplification that specifically matches the primers and probes of the PCR testing kit (summarised by amplification efficiency). | *High,*  *Moderate,*  *Low,*  *No* | Latent | Quantity of viral RNA in purified sample, amplification efficiency |  |
| **Specimen adequacy control (20)** | A Sample Adequacy Control (SAC) targets a single copy human gene that should be present in each specimen. The SAC confirms adequate patient sample has been collected and appropriate testing has occurred. | *Pass,*  *Fail* | Observable | Specimen quality, conditions of transport, extraction efficiency | (33) |
| **Amplification quality control (28)** | A nontarget nucleic acid sequence coamplified with the sample to measure the quality of the amplification process. | *Pass,*  *Fail* | Observable | Amplification efficiency, extraction efficiency | (36, 38) |
| **Detection Ct threshold (29)** | The number of cycles set within a PCR system determines when a fluorescent signal exceeds the background level indicating a positive sample. A higher threshold allows greater amplification, in turn allowing the system to be more sensitive. A low threshold may be 35 and a high threshold may be 40. The prior has been set to a 99% chance of using a high (and therefore more sensitive) Ct threshold. | *High,*  *Low* | Observable | None | (5, 13, 38) |
| **Shared target with non-targeted organism (9)** | The specificity of the SARS-CoV-2 nucleic acid sequence within the PCR assay to the SARS-CoV-2 within the sample tested. Prior distribution sets 99.9% to be false, however, if true, this (and this alone) introduces the risk of false positives. | *True,*  *False* | Observable | None | (35, 36) |
| **Detection of target (10)** | Detection of the nucleic acid sequence specific to the SARS-CoV-2 virus targeted by the PCR assay. | *True,*  *False* | Observable | Shared target with non-targeted organism,  quantity of target in amplified sample, detection Ct threshold | (39) |
| **Laboratory report (30)** | The reported result of the SARS-CoV-2 PCR assay. This is set to be a deterministic node that is Detected if detection of target was true; or, in the absence of detection, NotDetected if quality controls were passed and Indeterminate otherwise. | *Detected, NotDetected, Indeterminate* | Observable | Detection of target,  amplification control,  specimen quality control | (40, 41) |
| **Predicted classification (31)** | Model prediction expressing the predictive probability of a truly positive, truly negative, falsely positive, and falsely negative results. | *TrulyPos,*  *TrulyNeg,*  *FalselyPos*  *FalselyNeg* | Latent | Viral load at sample site, detection of target |  |

**References**

1. Luo L, Liu D, Liao X, Wu X, Jing Q, Zheng J, et al. Contact Settings and Risk for Transmission in 3410 Close Contacts of Patients With COVID-19 in Guangzhou, China : A Prospective Cohort Study. Ann Intern Med. 2020;0(0):null.

2. Metlay JP, Haas JS, Soltoff AE, Armstrong KA. Household Transmission of SARS-CoV-2. JAMA Network Open. 2021;4(2):e210304-e.

3. Heald-Sargent T, Muller WJ, Zheng X, Rippe J, Patel AB, Kociolek LK. Age-Related Differences in Nasopharyngeal Severe Acute Respiratory Syndrome Coronavirus 2 (SARS-CoV-2) Levels in Patients With Mild to Moderate Coronavirus Disease 2019 (COVID-19). JAMA Pediatrics. 2020;174(9):902-3.

4. Bi Q, Wu Y, Mei S, Ye C, Zou X, Zhang Z, et al. Epidemiology and Transmission of COVID-19 in Shenzhen China: Analysis of 391 cases and 1,286 of their close contacts. medRxiv. 2020.

5. Sethuraman N, Jeremiah SS, Ryo A. Interpreting Diagnostic Tests for SARS-CoV-2. JAMA. 2020;323(22):2249-51.

6. Wiersinga WJ, Rhodes A, Cheng AC, Peacock SJ, Prescott HC. Pathophysiology, Transmission, Diagnosis, and Treatment of Coronavirus Disease 2019 (COVID-19): A Review. JAMA. 2020;324(8):782-93.

7. Kucirka LM, Lauer SA, Laeyendecker O, Boon D, Lessler J. Variation in False-Negative Rate of Reverse Transcriptase Polymerase Chain Reaction–Based SARS-CoV-2 Tests by Time Since Exposure. Annals of Internal Medicine. 2020;173(4):262-7.

8. Lee S, Kim T, Lee E, Lee C, Kim H, Rhee H, et al. Clinical Course and Molecular Viral Shedding Among Asymptomatic and Symptomatic Patients With SARS-CoV-2 Infection in a Community Treatment Center in the Republic of Korea. JAMA Internal Medicine. 2020.

9. Caruana G, Croxatto A, Coste AT, Opota O, Lamoth F, Jaton K, et al. Diagnostic strategies for SARS-CoV-2 infection and interpretation of microbiological results. Clinical Microbiology and Infection. 2020;26(9):1178-82.

10. Sun Y, Feng, Y., Chen, J., Li, B., Luo, Z., & Wang, P. . Clinical Features of Fatalities in Patients With COVID-19. Disaster Medicine and Public Health Preparedness. 2020;1-3.

11. Yang Y, Yang M, Shen C, Wang F, Yuan J, Li J, et al. Evaluating the accuracy of different respiratory specimens in the laboratory diagnosis and monitoring the viral shedding of 2019-nCoV infections. medRxiv. 2020:2020.02.11.20021493.

12. Azzi L, Carcano G, Gianfagna F, Grossi P, Dalla Gasperina D, Genoni A, et al. Saliva is a reliable tool to detect SARS-CoV-2. Journal of Infection. 2020.

13. Liu Y, Liao W, Wan L, Xiang T, Zhang W. Correlation Between Relative Nasopharyngeal Virus RNA Load and Lymphocyte Count Disease Severity in Patients with COVID-19. Viral Immunology. 2020;0(0):null.

14. Pujadas E, Chaudhry F, McBride R, Richter F, Zhao S, Wajnberg A, et al. SARS-CoV-2 Viral Load Predicts COVID-19 Mortality. medRxiv. 2020.

15. To Kea. Temporal profiles of viral load in posterior oropharyngeal saliva samples and serum antibody responses during infection by SARS-CoV-2: an observational cohort study. The Lancet Infectious Diseases. 2020;20(5):565-74.

16. Pan Y, Zhang D, Yang P, Poon LL, Wang Q. Viral load of SARS-CoV-2 in clinical samples. The Lancet Infectious Diseases. 2020;20(4):411-2.

17. Garnett L, Bello A, Tran KN, Audet J, Leung A, Schiffman Z, et al. Comparison analysis of different swabs and transport mediums suitable for SARS-CoV-2 testing following shortages. J Virol Methods. 2020;285:113947-.

18. Centers for Disease Control Prevention. Interim guidelines for collecting, handling, and testing clinical specimens from persons under investigation (PUIs) for coronavirus disease 2019 (COVID-19) 2020 [updated 8th July 2020.

19. McCulloch DJ, Kim AE, Wilcox NC, Logue JK, Greninger AL, Englund JA, et al. Comparison of Unsupervised Home Self-collected Midnasal Swabs With Clinician-Collected Nasopharyngeal Swabs for Detection of SARS-CoV-2 Infection. JAMA Network Open. 2020;3(7):e2016382-e.

20. Tu Y-P, Jennings R, Hart B, Cangelosi GA, Wood RC, Wehber K, et al. Swabs Collected by Patients or Health Care Workers for SARS-CoV-2 Testing. New England Journal of Medicine. 2020;383(5):494-6.

21. Fang Y, Zhang H, Xie J, Lin M, Ying L, Pang P, et al. Sensitivity of Chest CT for COVID-19: Comparison to RT-PCR. Radiology. 2020;296(2):E115-E7.

22. Raschke RA, Curry SC, Glenn T, Gutierrez F, Iyengar S. A Bayesian Analysis of Strategies to Rule Out Coronavirus Disease 2019 (COVID-19) Using Reverse Transcriptase–Polymerase Chain Reaction. Archives of Pathology & Laboratory Medicine. 2020;144(8):915-6.

23. Wang W, Xu Y, Gao R, Lu R, Han K, Wu G, et al. Detection of SARS-CoV-2 in Different Types of Clinical Specimens. JAMA. 2020;323(18):1843-4.

24. Young BE, Ong S, Kalimuddin S, Low JG, Tan SY, Loh J, et al. Epidemiologic Features and Clinical Course of Patients Infected With SARS-CoV-2 in Singapore. JAMA. 2020;323(15):1488-94.

25. Wyllie AL, Fournier J, Casanovas-Massana A, Campbell M, Tokuyama M, Vijayakumar P, et al. Saliva or Nasopharyngeal Swab Specimens for Detection of SARS-CoV-2. New England Journal of Medicine. 2020.

26. Udugama B, Kadhiresan P, Kozlowski HN, Malekjahani A, Osborne M, Li VYC, et al. Diagnosing COVID-19: The Disease and Tools for Detection. ACS Nano. 2020;14(4):3822-35.

27. Ali N, Rampazzo RdCP, Costa ADT, Krieger MA. Current Nucleic Acid Extraction Methods and Their Implications to Point-of-Care Diagnosticsvol. BioMed Research International. 2017;2017:13 pages.

28. Hammerling JA. A Review of Medical Errors in Laboratory Diagnostics and Where We Are Today. Laboratory Medicine. 2012;43(2):41-4.

29. Osaro E, Chima N. Challenges of a negative work load and implications on morale, productivity and quality of service delivered in NHS laboratories in England. Asian Pac J Trop Biomed. 2014;4(6):421-9.

30. Broeders S, Huber I, Grohmann L, Berben G, Taverniers I, Mazzara M, et al. Guidelines for validation of qualitative real-time PCR methods. Trends in Food Science & Technology. 2014;37(2):115-26.

31. Sung H, Han M-G, Yoo C-K, Lee S-W, Chung Y-S, Park J-S, et al. Nationwide External Quality Assessment of SARS-CoV-2 Molecular Testing, South Korea. Emerging Infectious Diseases. 2020;26(10).

32. Schrader C, Schielke A, Ellerbroek L, Johne R. PCR inhibitors – occurrence, properties and removal. Journal of Applied Microbiology. 2012;113(5):1014-26.

33. Brukner I, Eintracht S, Papadakis AI, Faucher D, Lamontagne B, Spatz A, et al. Maximizing confidence in a negative result: Quantitative sample adequacy control. Journal of Infection and Public Health. 2020;13(7):991-3.

34. Vogels CBF, Brito AF, Wyllie AL, Fauver JR, Ott IM, Kalinich CC, et al. Analytical sensitivity and efficiency comparisons of SARS-COV-2 qRT-PCR primer-probe sets. medRxiv. 2020:2020.03.30.20048108.

35. Nalla AK, Casto AM, Huang M-LW, Perchetti GA, Sampoleo R, Shrestha L, et al. Comparative Performance of SARS-CoV-2 Detection Assays Using Seven Different Primer-Probe Sets and One Assay Kit. J Clin Microbiol. 2020;58(6):e00557-20.

36. Corman VM, Landt O, Kaiser M, Molenkamp R, Meijer A, Chu DK, et al. Detection of 2019 novel coronavirus (2019-nCoV) by real-time RT-PCR. Eurosurveillance. 2020;25(3):2000045.

37. Osório NS, Correia-Neves M. Implication of SARS-CoV-2 evolution in the sensitivity of RT-qPCR diagnostic assays. The Lancet Infectious Diseases. 2020.

38. Han MS, Byun J-H, Cho Y, Rim JH. RT-PCR for SARS-CoV-2: quantitative versus qualitative. The Lancet Infectious Diseases. 2020.

39. Espy MJ, Uhl JR, Sloan LM, Buckwalter SP, Jones MF, Vetter EA, et al. Real-time PCR in clinical microbiology: applications for routine laboratory testing. Clin Microbiol Rev. 2006;19(1):165-256.

40. Woloshin S, Patel N, Kesselheim AS. False Negative Tests for SARS-CoV-2 Infection — Challenges and Implications. New England Journal of Medicine. 2020;383(6):e38.

41. Watson J, Whiting PF, Brush JE. Interpreting a covid-19 test result. BMJ. 2020;369:m1808.
