# Supplementary material for: Bridging the gaps in test interpretation of SARS-CoV-2 through Bayesian network modelling
Source: Epidemiol Infect. 2021 Jun 23;149:e166. doi: 10.1017/S0950268821001357 (PMC8314199; doi:10.1017/S0950268821001357)

# Scenario 1

## (a) Heavy exposure
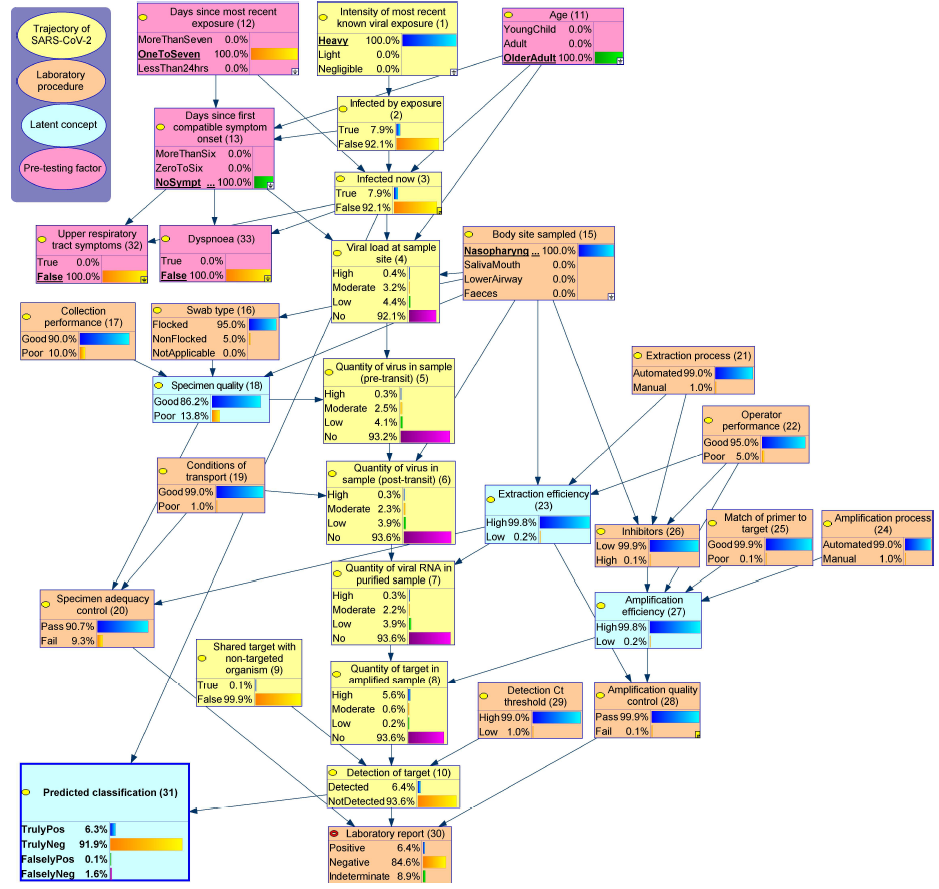


# Scenario 1

## (b) Heavy exposure with symptoms


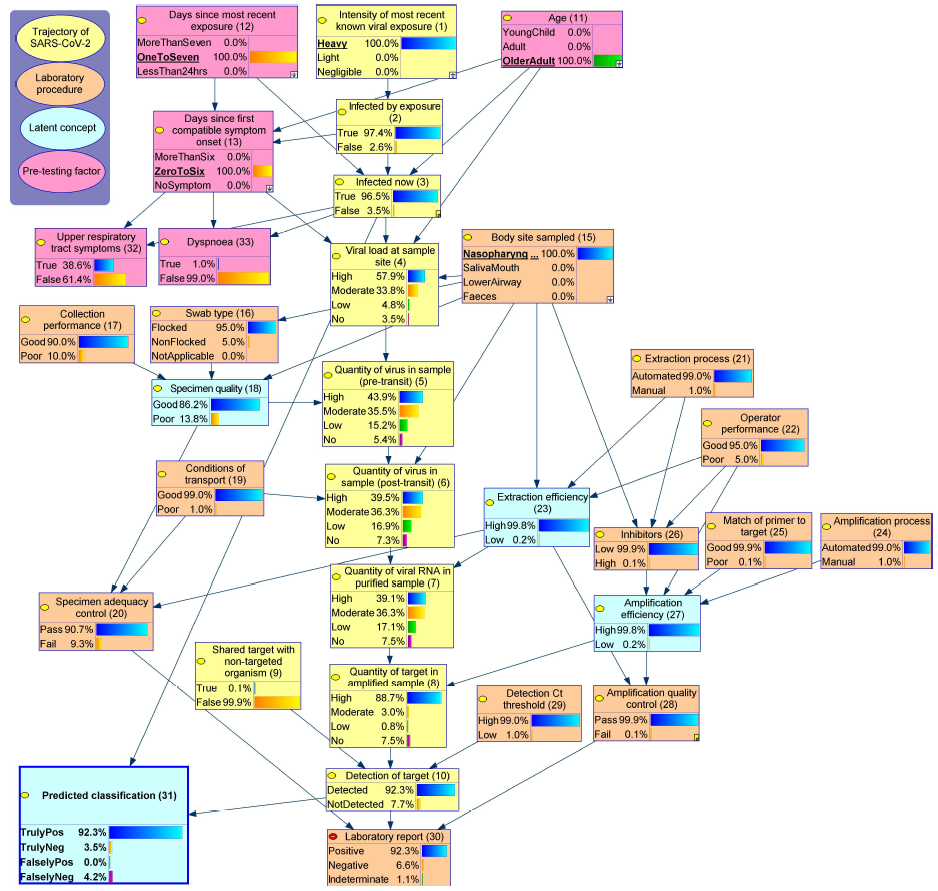


# Scenario 1

## (c) Light exposure


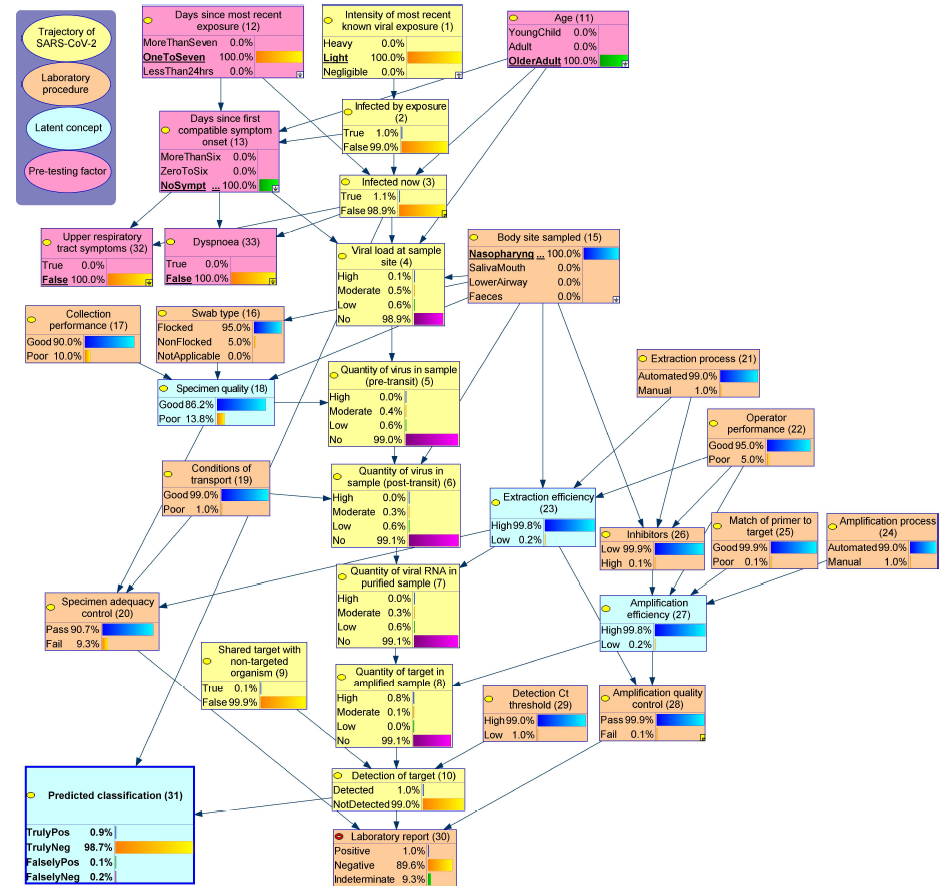


# Scenario 2

## (a) Good specimen quality


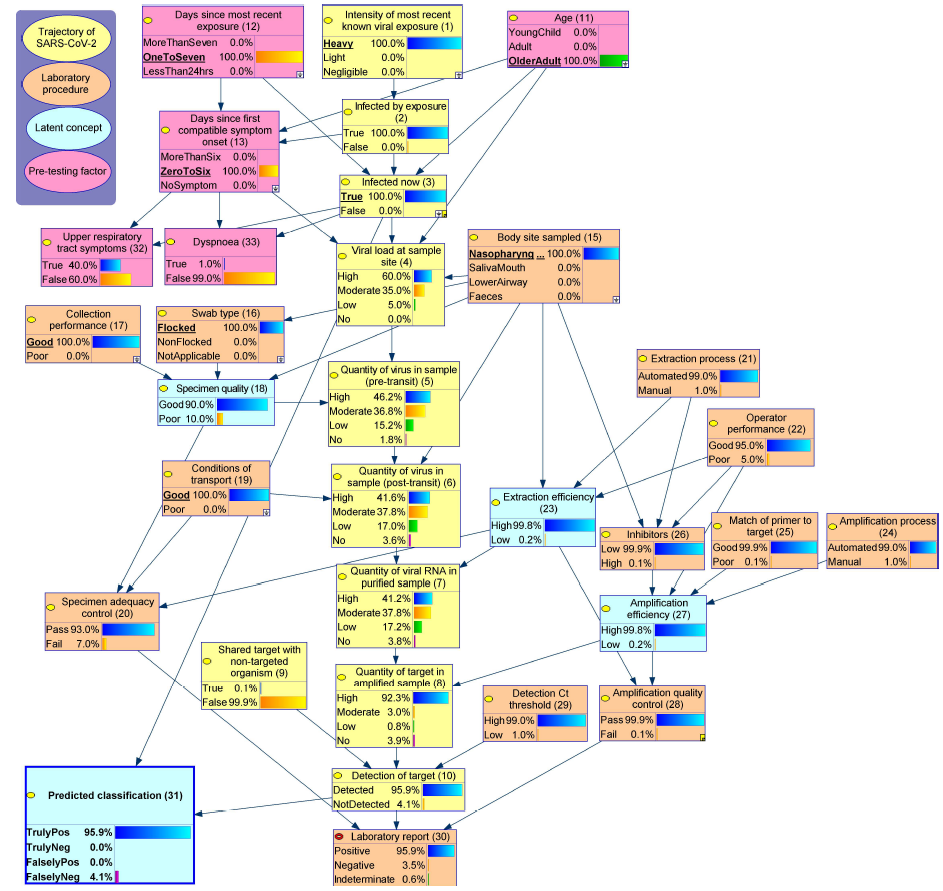


# Scenario 2

## (b) Poor specimen quality


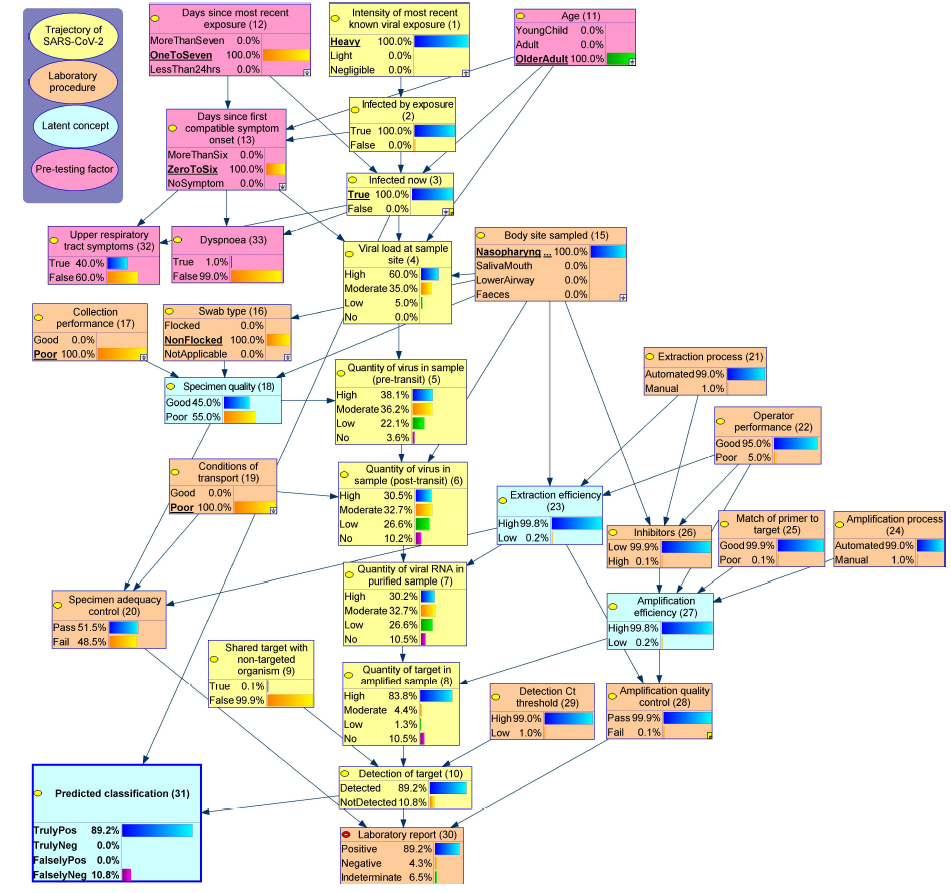


# Scenario 3

## (a) Infected tested negative
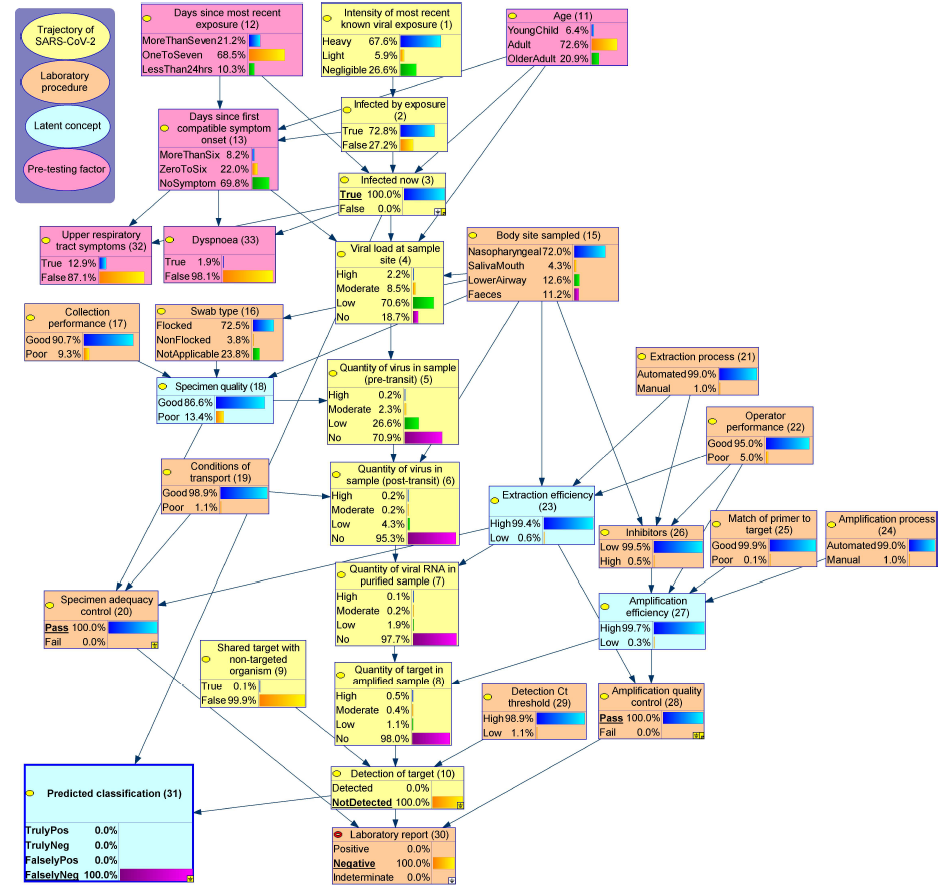


# Scenario 3

## (b) Infected tested positive


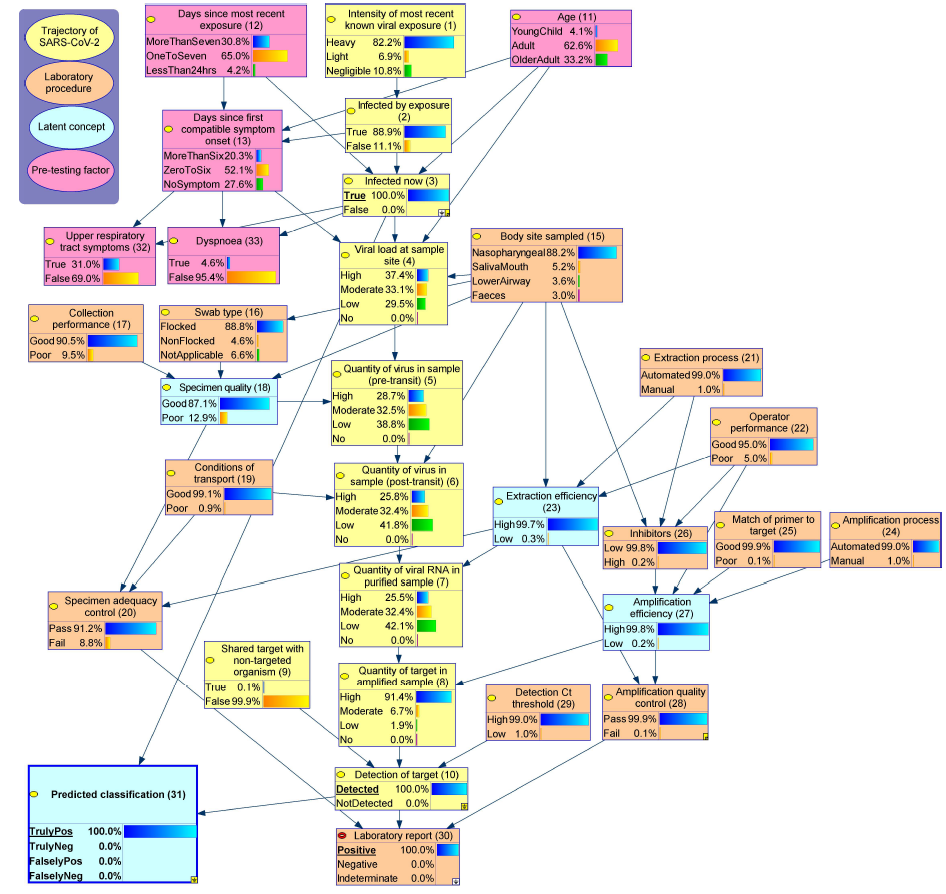


# Scenario 4

## (a) Low prevalence with upper respiratory tract symptoms


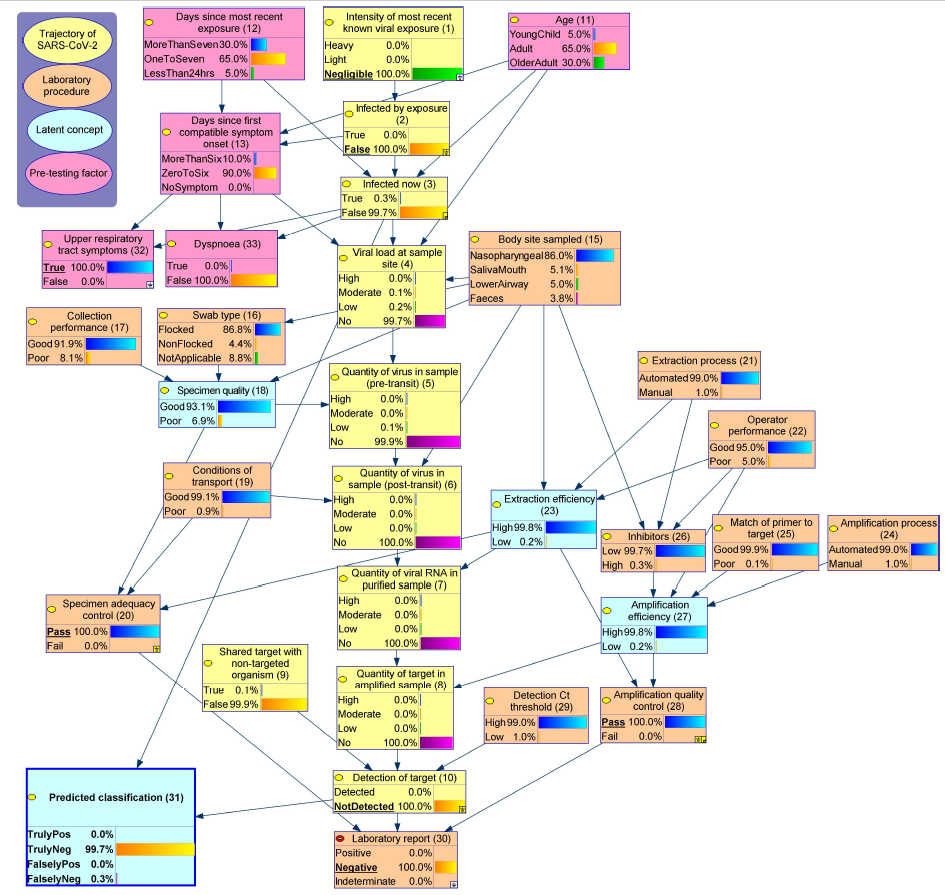


# Scenario 4

## (b) High prevalence with upper respiratory tract symptoms


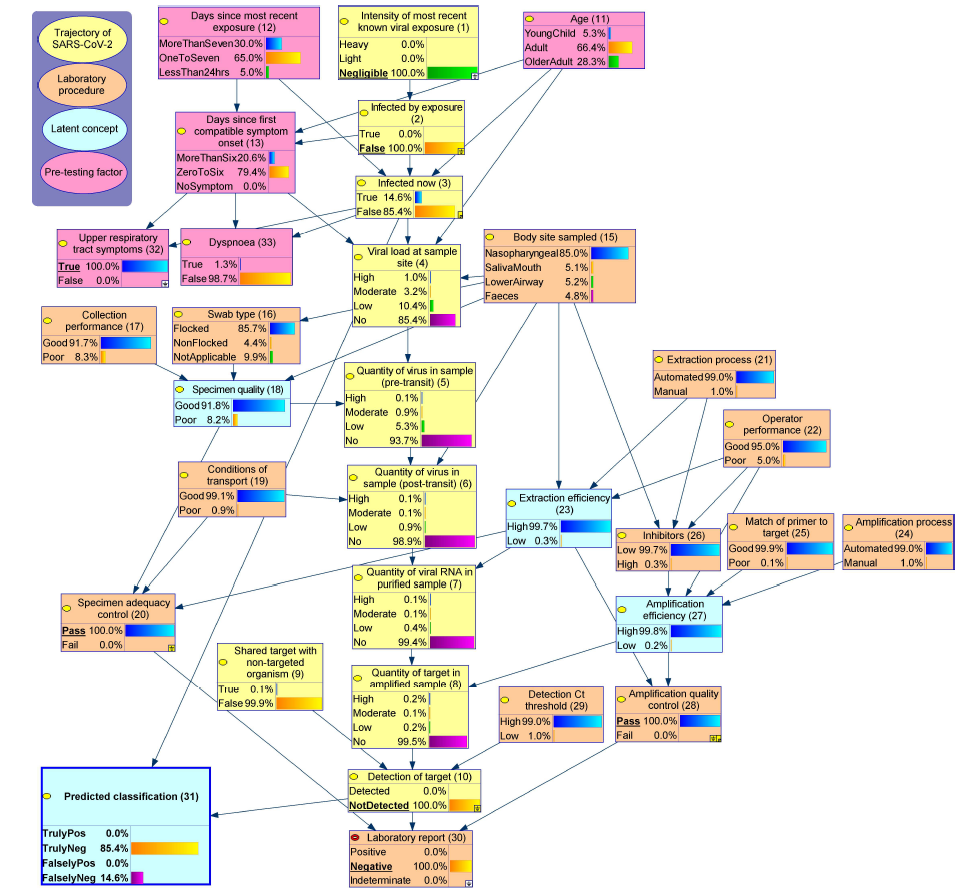


# Scenario 4

## (c) High prevalence with dyspnoea


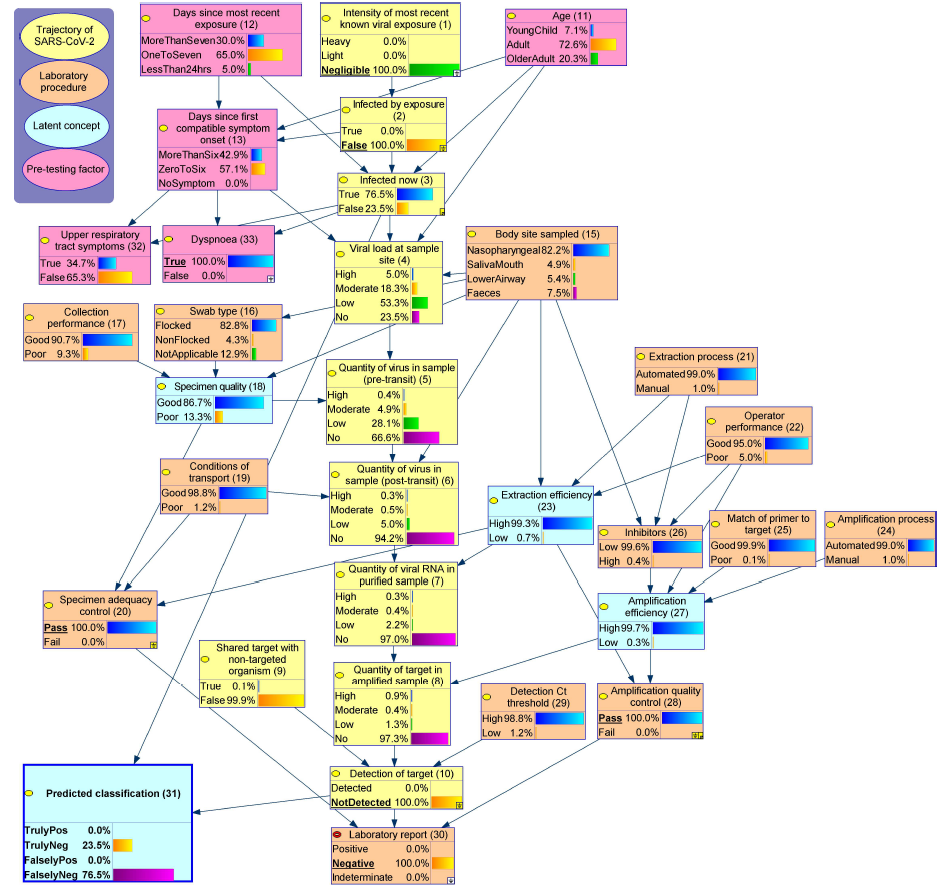

Supplement: Supplementary file 1 [file hygsup.zip › S0950268821001357sup002.docx]
